# Immunological Responses of Arsenicum album 30CH to Combat COVID-19: Protocol for a Double-Blind, Randomized, Placebo-Controlled Clinical Trial in the Pathanamthitta District of Kerala

**DOI:** 10.2196/48479

**Published:** 2023-10-16

**Authors:** Panaparambil Azis Suhana, Lata Kusum, Jain Vij Shruti, Gopinathan Pillai Sreekanth, Damodaran Bijukumar, R T Shaji Kumar, K C Muraleedharan, Subhash Kaushik

**Affiliations:** 1 Department of Clinical Research Central Council for Research in Homoeopathy New Delhi India; 2 Department of Applied Biology, Council of Scientific and Industrial Research-Indian Institute of Chemical Technology Hyderabad India; 3 District Medical Office (Pathanamthitta) Directorate of Homoeopathy Government of Kerala Pathanamthitta India; 4 National Homoeopathy Research Institute Mental Health Central Council for Research in Homoeopathy Government of India Kottayam India

**Keywords:** COVID-19, Arsenicum album 30CH, randomized controlled trial, Pathanamthitta, immunology

## Abstract

**Background:**

COVID-19 is a recent major public health concern caused by the SARS-CoV-2 virus, with approximately 44.6 million COVID-19–positive cases and 530,000 deaths in India (as of February 1, 2023). The COVID-19 vaccination drive in India was initiated in January 2021; however, an effective preventive strategy with high efficacy and immunological safety remains elusive.

**Objective:**

The aim of this study is to assess the immunogenic responses of *Arsenicum album* 30CH (AA30CH) as COVID-19 prophylaxis, including assessment of immunological markers, innate and acquired immune responses, COVID-19 symptoms, and its associated antibody responses.

**Methods:**

This randomized controlled clinical trial (RCT) will include two parallel comparator groups of AA30CH and placebo with an allocation ratio of 1:1 conducted in the Pathanamthitta district of Kerala, India. The placebo or AA30CH will be administered in three intervention schedules and blood samples will be collected before and after each of the intervention schedules. Based on the inclusion and exclusion criteria, 112 participants per arm (with an expected dropout of 20%) will be screened. Immunogenic responses will be evaluated by determining the antigen density and modulation in immunological markers and lymphocyte subsets CD3, CD4, CD8, CD24, CD27, CD38, CD4 interferon-γ, CD4 CD17, CD4 CD25 (activated T lymphocytes), T cells, B cells, dendritic cells (mature and immature), and natural killer cells on days 1, 5, 23,27, 45, 49, and 66. The innate and acquired immune responses will also be evaluated by a real-time reverse-transcriptase polymerase chain reaction (RT-PCR) array profiler (84-gene set) before and after the study interventions. The toxicity status of AA30CH in study participants will be evaluated through hepatic, renal, and hematological parameters and peripheral smears on days 1, 5, 23, 27, 45, 49, and 66. The number of participants developing COVID-19–like symptoms per National Centre for Disease Control guidelines and the number of participants testing positive for COVID-19 in RT-PCR during follow-ups in any of the three intervention schedules will be identified. Moreover, a subgroup analysis will be used to assess the COVID-19 antibody responses between vaccinated and unvaccinated participants.

**Results:**

This RCT protocol has been approved by various committees and funded by the Central Council for Research in Homoeopathy, Ministry of Ayush, Government of India. The project has been implemented in collaboration with the Department of Homoeopathy, Government of Kerala. The RCT was rolled out on January 25, 2023, and enrollment was completed April 3, 2023. The immunological assays will be conducted at the Department of Biotechnology-Translational Health Science and Technology Institute, Faridabad, India.

**Conclusions:**

This study will represent the first evaluation of the immunological efficacy and safety of AA30CH in an RCT, which may significantly impact the use of homeopathy as an evidence-based medicine approach.

**Trial Registration:**

Clinical Trials Registry-India CTRI/2022/08/045089; https://tinyurl.com/mryrpkvk

**International Registered Report Identifier (IRRID):**

DERR1-10.2196/48479

## Introduction

COVID-19 infection caused by SARS-CoV-2 is a recent major public health burden leading to clusters of mild to moderate respiratory illnesses. COVID-19 became a pandemic affecting more than 200 countries worldwide owing to its high infectivity [1,2]. COVID-19 infection is accompanied by an aggressive inflammatory response that releases several proinflammatory cytokines [3,4]. The host immune response to the SARS-CoV-2 virus is hyperactive, resulting in an excessive inflammatory reaction [5]. The incubation period of COVID-19 was reported to be 2-14 days [6,7]. It is currently believed that SARS-CoV-2 is most likely of zoonotic origin [8-10] and transmits from person to person by infected droplets, with more severe infections mainly found in older people and those suffering from comorbidities [11,12].

The World Health Organization (WHO) dashboard of COVID-19 reported approximately 753,479,439 confirmed cases of COVID-19, including 6,812,798 confirmed deaths (as of January 31, 2023) globally. The United States has been declared the country with the highest number of cases, followed by China, India, France, Germany, Brazil, Japan, the Republic of Korea, Italy, the United Kingdom, the Russian Federation, Turkey, and Spain, respectively (as of January 31, 2023) [13]. As per official reports of the Government of India, the country reported 44,682,895 COVID-19–positive cases with 530,740 deaths as of February 1, 2023. The Indian states of Maharashtra and Kerala reported the highest number of cases. Maximum deaths were reported in the second wave of COVID-19, which peaked in May 2021 [14]. The Government of India initiated the COVID-19 vaccination drive in January 2021. Nevertheless, an effective preventive measure needs to be identified with evidence of breakthrough infections and reduced protection against the Delta variant of SARS-CoV-2.

Several therapeutic strategies and clinical evaluations are ongoing to combat SARS-CoV-2 infection; however, no effective treatment has been developed with immunological safety. The WHO initiated a large global, randomized clinical study on four therapies (remdesivir, hydroxychloroquine, lopinavir/ritonavir, and interferon [IFN] therapy), showing that all four therapies had little or no influence on overall mortality, ventilation initiation, or hospital stay for hospitalized patients [15]. Furthermore, the usage of antibiotics in patients with COVID-19 raises the risk of harmful consequences such as antibiotic resistance [16,17].

Homeopathy has been vital in preventing epidemics, especially before modern sanitation, antibiotics, and vaccinations [18]. Homeopathy has also displayed effectiveness in combating many outbreaks in its history of 200 years, such as scarlet fever in 1799 [19], leptospirosis in 2007 [20], and dengue fever in 2018 [21,22]. Homeopathy has also been successfully documented in the medical literature for both the treatment and prophylactic use of flu-like epidemics such as the “Spanish” flu of 1918 [23], “Asian” flu of 1951 [24], and H_1_N_1_ influenza pandemic of 2009-2010 [25]. The homeopathic formulation *Arsenicum album* 30CH (AA30CH) was previously reported to be effective in various respiratory diseases such as swine flu, influenza-like illness, and other respiratory illnesses [25-29]. In one study, *Arsenicum album*, as one of the constituents of 7X-24X complex, exhibited nuclear factor-κB hyperactivity according to reduced expression of the reporter gene in green fluorescent protein-transfected HT29 cells and also reduced the release of tumor necrosis factor-α in macrophages [30]. Due to the looming threat of the COVID-19 pandemic and the lack of effective treatments, alternative preventive strategies need to be established.

During the pandemic, AA30CH was advised as a preventative medicine for COVID-19 by the Ministry of Ayush, Government of India, toward the end of January 2020 [31]. A subsequent advisory issued by the Ministry of Ayush on March 6, 2020, also mentions the immunomodulatory action of AA30CH [32]. However, the exact role of AA30CH in innate immunity against COVID-19 was not fully understood. The observation that individuals with underlying diseases are more susceptible to severe illness than healthy people or young children is mainly attributed to the low efficacy of the innate immune response [33]. Therefore, the aim of this study is to investigate the cellular pathways related to innate and adaptive responses to AA30CH using flow cytometry–based immunological assays and gene expression analysis of 84 vital genes with a real-time reverse transcription-polymerase chain reaction (RT-PCR) array profiler. This PCR array profiler includes genes related to the interleukin-1 receptor (IL-1R) and Toll-like receptor (TLR) signaling pathways, including IL-1R and TLR genes involved in the sensing of pathogens. Genes related to host defense are also represented in this array, including the acute-phase response, complement activation, the inflammatory response, and the antiviral humoral response.

Moreover, gene expression profiling of human natural killer (NK) cell responses will also be investigated. This randomized controlled clinical trial (RCT) is being conducted by the Central Council for Research in Homoeopathy (CCRH), an apex research body under the Ministry of Ayush, Government of India, in the Pathanamthitta district of Kerala state, in close collaboration with the District Medical Office (under the Department of Homoeopathy, Government of Kerala) after signing an agreement with the Government of Kerala. The CCRH also signed a Memorandum of Understanding with the Department of Biotechnology-Translational Health Science and Technology Institute (DBT-THSTI), Faridabad, a Coalition for Epidemic Preparedness Initiative–approved laboratory facility under the Government of India for conducting the immunological assays as part of the primary objective of the study.

The primary objectives of the study are to elucidate the immunogenic responses of AA30CH in COVID-19 prophylaxis by determining the antigen density and identifying the variation in immunological markers and lymphocyte subsets CD3, CD4, CD8, CD24, CD27, CD-38, CD4 IFN-γ, CD4 CD17, CD4 CD25 (activated T lymphocytes), T cells, B cells, dendritic cells (DCs; mature and immature), and NK cells; and to estimate the innate and acquired immune responses by real-time RT-PCR array analysis (see Multimedia Appendix 1). The secondary objectives of the study are the toxicological evaluation in study participants who took either a placebo or AA30CH, limited to hepatic, renal, and hematological parameters, by conducting liver function tests (LFTs), renal function tests (RFTs), complete blood count (CBC), and peripheral smear; to identify the number of participants developing COVID-19–like symptoms as per National Centre for Disease Control (NCDC) guidelines during follow-ups in any of the three intervention schedules; to identify the number of participants testing positive for COVID-19 in RT-PCR during follow-ups in any of the three intervention schedules; and to determine the COVID-19 antibody responses between vaccinated and unvaccinated participants.

## Methods

### Study Design

**Figure 1:**
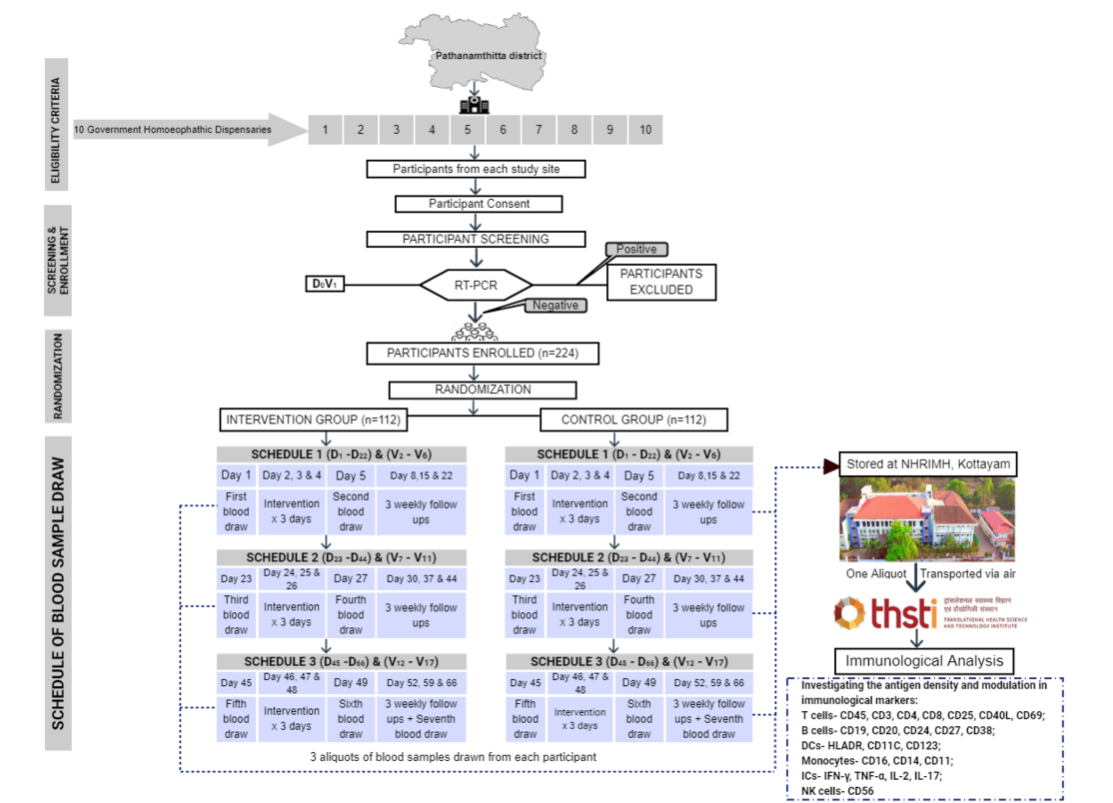
Flow diagram of the screening and randomization of the participants. D: day; DC: dendritic cell; IFN: interferon; IL: interleukin; NK: natural killer; NHRIMH: National Homoeopathy Research Institute in Mental Health; RT-PCR: reverse transcription-polymerase chain reaction; TNF: tumor necrosis factor; V: visit.

This clinical trial is designed as a randomized, double-blind, placebo-controlled clinical study with two parallel comparator arms of AA30CH and placebo, using an allocation ratio of 1:1. The participants will be enrolled and randomized only after two phases of initial screening (screenings I and II), followed by three schedules of intervention and follow-ups in the intervention phase of 66 days. The flow chart of the study design is represented in Figure 1. The protocol is reported following the SPIRIT (Standard Protocol Items: Recommendations for Interventional Trials) 2013 guidelines.

### Study Setting

This RCT will be implemented in the 10 government homeopathic dispensaries/hospitals located in 10 different panchayats of the state of Kerala in India, identified by the District Medical Officer (Pathanamthitta District), Department of Homoeopathy, Government of Kerala, based on the higher incidence of COVID-19 and elevated test positivity rate (TPR). These identified study sites are: (1) Government Homoeopathic Dispensary, Elanthoor panchayat; (2) Government Homoeopathic Dispensary, Naranganam panchayat; (3) Government Homoeopathic Dispensary, Kulanada panchayat; (4) Government Homoeopathic Dispensary, Pandalam panchayat; (5) Government Homoeopathic Dispensary, Ezhamkulam panchayat; (6) Government Homoeopathic Dispensary, Puthusserimala Ranni panchayat; (7) Government Homoeopathic Dispensary, Mezhuveli panchayat; (8) Ayush Primary Health Centre Homoeopathy (under National Health Mission, Government of India), Ranni Angadi panchayat; (9) Government Homoeopathic Dispensary, Ayush Health and Wellness Center, Kuttappuzha panchayat; and (10) Government Homoeopathic Hospital, Kottanad panchayat. The National Homoeopathy Research Institute in Mental Health (NHRIMH), located in the Kottayam district of Kerala state under the CCRH, Government of India, is designated as the facilitating institute of the study with prior approvals, and two nodal site principal investigators (PIs) and supporting team will operate from the NHRIMH during the RCT. The PI will form monitoring teams who will visit the 10 study sites on all blood draws and on intervention days of the trial and report to the competent authorities in real time.

### Eligibility Criteria

#### Inclusion Criteria

Apparently healthy male and female participants, aged 18-60 years, residents of the Pathanamthitta district of Kerala, with negative COVID-19 RT-PCR test reports at least 48 hours before the first day of the intervention, and those who have not taken *Arsenicum album* medicine in any homeopathic potencies in the past 4 months will be included in the study; both COVID-19 vaccinated and unvaccinated participants will be eligible for inclusion.

#### Exclusion Criteria

Participants with a positive COVID-19 RT-PCR test report at least 48 hours before the first day of the intervention; those with history of severe viral diseases in the past 3 months, such as herpes zoster, dengue, chikungunya, and chicken pox; those having a history of chronic respiratory ailments with shortness of breath as a recurring symptom; and those taking concomitant medications such as immunosuppressants, hormonal therapies, or Ayush therapies for chronic ailments will be excluded from the study.

### Sample Size

The study is aimed at evaluating the immunologic responses of AA30CH. The study is based on earlier encouraging leads provided through community studies on the role of AA30CH in preventing COVID-19. Several parameters are in consideration for the evaluation of immunogenic responses. With regard to the computation of sample size, based on the literature and expert opinions, it is proposed to consider some limited immunologic parameters that are more relevant. Owing to the scarce availability of literature, it would be quite challenging to hypothesize the assumption. Assuming a type 1 error of 5% and a power of 80%, it was estimated that there would be 93 participants each in the intervention and control groups, with a total of 186 participants. This could be escalated based on attrition due to loss in follow-up. If this loss to follow-up is considered at 20%, a total of 112 participants have to be enrolled in each group, with a total sample of 224 that would need to be recruited for the study.

### Randomization and Blinding

The study participants will be recruited from 10 preidentified study sites in the Pathanamthitta district of Kerala. Recruitment will be achieved according to the study inclusion criteria and based on the initial and final screening. The participants will be allocated to either intervention or placebo groups as per the randomization sequence generated using PASS 2021 (v21.03) software. Prenumbered or coded identical containers will be used for allocation concealment. The PI at CCRH headquarters will be involved in the sequence generation process and allocation concealment procedure. The PI will label the interventions as per the randomization codes, which will be handed over to the site PIs. The site PIs will enroll participants and randomly assign participants to interventions. The trial participants and the site PIs assigning participants to interventions will be blinded. To ensure blinding quality, the packaging for both the intervention and placebo will be identical in shape, color, size, taste, and odor. The unblinding will be done only at the end of the study by the PI at CCRH headquarters. If the participants reported severe adverse events (SAEs) during the trial, unblinding may be performed per Data and Safety Monitoring Board (DSMB) or ethical committee recommendations. The reason for urgent unblinding should be well-documented.

### Pretrial Preparation

The selection of 10 homeopathic dispensaries was based on the COVID-19 incidences and TPR in the Pathanamthitta district. The CCRH conducted a preliminary short survey to identify these 10 sites. Following Central Ethics Committee approval, the clinical trial was registered in the Clinical Trials Registry-India (CTRI) under registration number CTRI/2022/08/045089 (August 30, 2022). An Indian Council of Medical Research–approved and National Accreditation Board for Testing and Calibration Laboratories–accredited laboratory was engaged for RT-PCR screening, hematology, clinical biochemistry, and peripheral blood mononuclear cells (PBMCs) isolation. The isolated PBMC samples for conducting immunological assays will be stored in a –80°C freezer located at the central laboratory facility in the facilitating institute of the RCT. The isolated PBMC samples will be transported to the DBT-THSTI in batches for the immunological assays. The blood samples collected from the study participants will also be used to evaluate the safety profile of the drug, mainly in terms of hematology parameters, liver function, and renal function. Training will be administered to the laboratory staff for the procedures of PBMC isolation. A mock drill will also be conducted at one of the study sites to identify the practical difficulties encountered during the screening, participant recruitment, sample collection, and storage procedures (no participant recruitment will be allowed with the mock drill). All site PIs in the 10 sites will be included in the mock drill for the practical demonstration of handling the samples and to streamline the logistics involved in the sample transportation and PBMC isolation procedures until storage in the –80°C freezer. Detailed standard operating procedures for the procedures and data collection, case record forms (CRFs), and screening forms were designed and printed as individual booklets.

### Screening and Enrollment

All the participants will be voluntarily invited to the study and then required to sign an informed consent form containing information on the regulatory authorities and related procedures, including initial RT-PCR screening, laboratory screening, and subsequent randomization after enrollment. The RT-PCR test will screen the participants fulfilling the eligibility criteria for an ongoing COVID-19 infection. If the RT-PCR test result is found to be negative for COVID-19, the participant will be investigated for an initial (baseline) LFT, RFT, CBC, and peripheral blood smear as part of the second screening for any abnormal parameters. Those with borderline elevated or normal values will be enrolled in the trial, followed by randomization. Screened participants with positive RT-PCR reports for COVID-19 will be notified to the local health authorities. Their health status will be telephonically monitored during quarantine or hospital stay by the site PI. Each study site will maintain a log register that records all details of the screened participants and the reason(s) for exclusion. Enrolled participants will then be randomized and allocated to the intervention or the placebo arm as per the generated randomization sequence using PASS software version 21.03.

### Assessment, Data Analysis, and Manuscript Preparation

The laboratory parameters recorded during the seven blood draws of each participant will be analyzed for any statistically significant changes using appropriate statistical tests. The PBMCs isolated during the seven blood draws and stored in the –80°C freezer will be transported as air freight in liquid nitrogen tankers to DBT-THSTI, Faridabad, for immunological assays and data analysis. The interim reports will be confidentially submitted to the designated DSMB, internal committees of CCRH, and Ministry of Ayush, Government of India, on availability as per requirements. Manuscripts with the study findings will be prepared and submitted to Scopus-indexed peer-reviewed journals. Patents pertaining to this RCT will be filed as per the directions of the Ministry of Ayush, Government of India.

### Dropout, Withdrawal, and Suspension Criteria

#### Dropout Criteria

Participants who develop SAEs will be withdrawn from the RCT after reporting to the Institutional Ethics Committee. Participants who develop concomitant diseases of viral origin during the trial that would affect the efficacy and safety assessment intervention, participants with poor compliance, those changing the intervention or adding therapy prohibited under the study for any reason, breaking the double-blind setting of the clinical trial, and those found to have any serious violations of the inclusion/exclusion criteria after enrollment will be withdrawn from the study.

#### Voluntary Withdrawal of Participants

The participant can become unwilling/unable to continue in the RCT and request the site PI to withdraw them from the RCT; other participants who stop visiting even after prompt reminders by the site PI will be withdrawn from the study. Every participant can withdraw from the trial anytime for any reason and without prejudice. Any participant who withdraws from the trial shall undergo a complete final examination, if possible, or at least a last telephonic interview at the time of withdrawal from the study about the state of their health by the investigator, and if necessary, from the point of view of drug safety and the validity of study results. The reason for the withdrawal will be recorded in the Participant Off Study form of the CRF.

#### Conditions for Trial Suspension

If any SAE occurs during the study, the trial will be terminated immediately as per recommendations of the DSMB and Ethics Committee. If there is a major error in the trial or a serious protocol deviation during implementation, which would make it difficult to evaluate the drug efficacy and safety, the trial will be stopped. The trial will also be terminated if the funding agency or collaborating institution requests to stop the trial or if the regulatory authority cancels the trial. The Council is entitled to terminate the trial prematurely at any time for medical and/or organizational reasons. This decision will generally be made based on the recommendations of the Scientific Advisory Board, CCRH, New Delhi, or the Director of Homoeopathy, Government of Kerala.

### Intervention Procedure

#### Preparation of Study Intervention and Placebo Materials

AA30CH will be prepared at Dr Willmar Schwabe India Pvt Ltd, Noida, Uttar Pradesh, India (a licensed homeopathic drugs manufacturing company complying with Good Manufacturing Practice standards in India) as per the Homoeopathic Pharmacopoeia of India standards, using the active component of *Arsenicum album* in a fine powder mixed with glycerin, purified water, and pharmaceutical-grade ethanol following standard homeopathic drug manufacturing protocols approved by drug licensing authorities in India. The placebo (30CH) will be identically prepared using only glycerin, purified water, and pharmaceutical-grade ethanol, without the medicinal active component (*Arsenicum album*).

#### Interventions in Each Group

Following the second screening, participants with normal or borderline elevated values will be allocated to intervention and placebo arms as per the randomization sequence generated using the software PASS 2021 (v21.03). The intervention period for both groups consists of three schedules of 21 days each, with a total trial period of 66 days, including intervention, blood sample collection before and after the intervention, and three weekly telephonic follow-ups in each of the schedules. The sealed bottles of AA30CH and placebo 30CH dilutions will be opened at the in-house pharmacy of NHRIMH, Kottayam, under the supervision of the PI and the pharmacy in charge. Following standard pharmacy practice, these dilutions will further be used (approximately nine drops) for medicating one-grain lactose/sucrose (inert nonmedicated) tablets. Participants will be administered either the homeopathic medicine AA30CH or placebo as per the randomization sequence in three doses of single tablets orally for each of the 3 consecutive days of the 21-day schedule. Each of the three intervention schedules will be preceded and followed by a blood draw of the participants. The administered dose will be recorded in the Drug Dispensation Log of the CRF.

#### Criteria for Discontinuing or Modifying Allocated Interventions

All participants shall be advised to telephonically inform the site PI if they develop symptoms similar to COVID-19 infection. If any participant reports symptoms, the intervention shall be stopped, and if testing positive for COVID-19 in RT-PCR, the local health authorities will be informed by the site PI. Study participants shall be advised to take the treatment of their choice from the dispensary at the study site through teleconsultation or from other medical facilities nearby. In case of requirement, the study participants will also be provided support through an already allocated medical health insurance policy (valid for 1 year) for their hospitalizations or other related treatments. They shall be under observation in this study until their recovery from the symptoms and may report any events that may be an SAE. The site PI will record the days to recovery, disease stage, and severity of symptoms, among other clinical data. The details of treatment and progress will be acquired from family members or the participants themselves, which may be recorded in the follow-up and participant study forms in the CRF. A COVID-19–positive status with the intervention or placebo will be considered as the study endpoint for participants with a positive test result on RT-PCR.

#### Strategies to Improve Adherence to Intervention Protocols and Procedures for Monitoring Adherence

The participants must visit the study site for the 3 days of the intervention at each schedule. To ensure compliance, the independent dispenser will only administer the medicine and placebo to the participants at the prescribed dosage. The intervention procedure will be under the supervision of the site PI to ensure adherence. Any deviation in these procedures will be recorded in the Deviation/Unanticipated Problem Tracking Log of the CRF. Participants will be under observation of the site PI at the study site for at least 1 hour to monitor for any immediate drug-related adverse effects, which will be recorded and informed to the PI. If the participant feels any discomfort, they will be medically examined by the site PI. Vital parameters such as heart rate, respiratory rate, and blood pressure will be monitored and recorded. In case of any SAE, the participant will be referred to a tertiary-care facility after receiving appropriate first aid at the site by the site PI. The DSMB and Ethics Committee will be duly informed about these events. Blood samples will be drawn and stored 1 day before and after 3 days of the intervention and on the last day of the study by the designated laboratory involved in sample collection and storage. During each visit, participants will be compensated for travel from their home to the study site and provided with protein-based refreshments to minimize their difficulty at each visit to the study site, thereby improving adherence.

#### Relevant Concomitant Care and Interventions Permitted or Prohibited During the Trial

Any medical problem a participant faces during the trial period will be informed to and managed by the site PI of the study site appropriately and documented and reported to the PI. The administration of vaccinations is not permitted during the study duration as it would interfere with the study results. If a participant is taking immunosuppressants, hormonal therapies, or any other Ayush therapies for chronic ailments, they will be considered a dropout. If the participant reports the intake of any other concomitant medications not specified in the exclusion criteria, the same may be documented in the Concomitant Medication Log of the CRF.

### Outcome Measures

The primary outcome measures of the study are the estimation of antigen density and modulation in immunological markers (CD3, CD4, CD8, CD24, CD27, CD38, CD4 IFN-γ, CD4 CD17, CD4 CD25 [activated T lymphocytes], T cells, B cells, DCs [mature and immature], and NK cells) observed from the blood sample drawn 1 day before and after 3 days of the intervention (ie, on days 1, 5, 23,27, 45, and 49) and at the end of the study (day 66), along with the innate and acquired immune responses from the blood sample drawn on days 1, 23, 45, and 66 of the study interventions.

The secondary outcome measures of the study are to evaluate the toxicity status of study participants who have taken AA30CH or placebo based on hepatic, renal, and hematological parameters and peripheral smears 1 day before and after 3 days of the intervention (ie, on days 1, 5, 23, 27, 45, and 49) and at the end of the study (day 66); the number of participants developing COVID-19–like symptoms as per NCDC guidelines during follow-ups in any of the three intervention schedules; and the number of participants with COVID-19–like symptoms who test positive for RT-PCR during follow-ups in any of the three intervention schedules.

### Participant Timeline

**Figure 2:**
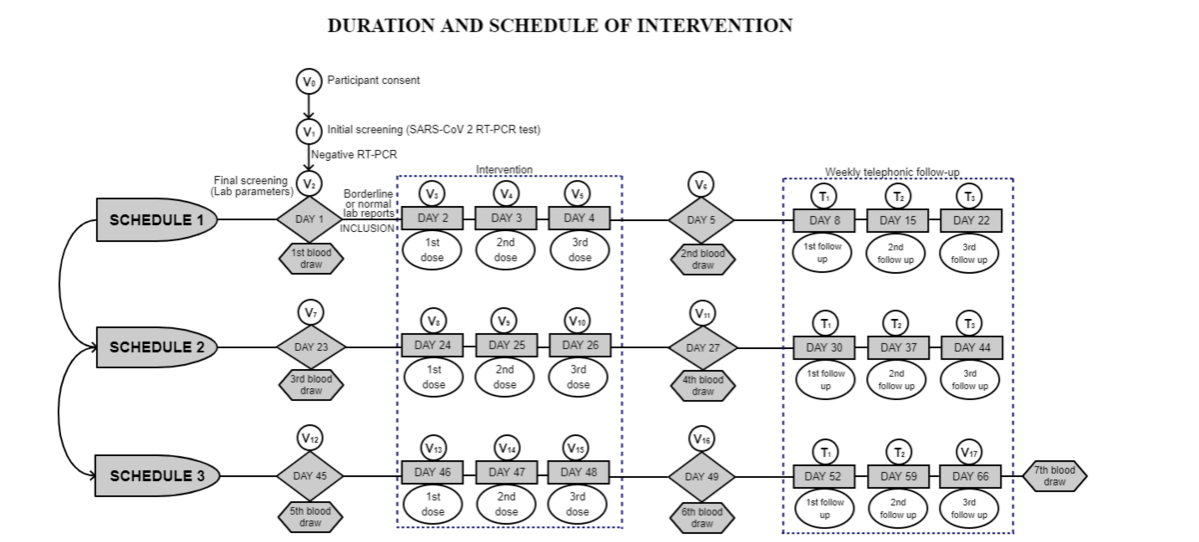
Flow diagram of three schedules of intervention (21 days each). RT-PCR: reverse transcription-polymerase chain reaction; T: telephonic follow-up; V: visit.

The study consists of three schedules of 21 days each. Each participant will have seven blood draw visits (V2, V6, V7, V11, V12, V16, and V17) during the study period. The blood samples will be drawn for conducting LFTs, RFTs, CBC, and peripheral smears 1 day before and after 3 days of the intervention (ie, on days 1, 5, 23, 27, 45, and 49) and at the end of the study (day 66) (Table 1, Figure 2).

**Table 1:** Participant visits (V) for the three schedules with the telephonic follow-up (T) timeline.

Procedures	Baseline		Schedule 1					Schedule 2					Schedule 3			
	V0	V1	V2	V3-V5	V6	T1-T3	V7		V8-V10	V11	T1-T3	V12		V13-15	V16	T1, T2, V17
Consent	✓															
RT-PCR^a^ test		✓														
Enrollment, randomization, and first intervention				✓^b^												
Blood sample collection for immunological assays (PBMCs^c^), innate and acquired immune responses, LFT^d^, RFT^e^, CBC^f^, peripheral smear			✓		✓		✓			✓		✓			✓	✓
Intervention (AA30CH^g^ or placebo)				✓					✓					✓		
Follow-ups						✓					✓					✓

^a^RT-PCR: reverse transcription-polymerase chain reaction.

^b^At V3 only.

^c^PBMC: peripheral blood mononuclear cell.

^d^LFT: liver function test.

^e^RFT: renal function test.

^f^CBC: complete blood count.

^g^AA30CH: *Arsenicum album* 30CH.

### Adverse Events

Any SAE that occurs during and after the study period and is possibly considered due to the study intervention or participation will be recorded and reported immediately to the concerned ethical committee and DSMB.

### Data Monitoring and Management

The PI will monitor the study regularly, and site visits/online meetings will be held periodically on intervention and blood draw days. The PI will allocate adequate time for such monitoring activities and ensure that the monitor or other compliance or quality assurance reviewer can access all the study-related documents and study-related facilities (eg, pharmacy, diagnostic laboratory). The original records of participants (including CRFs, consent forms, follow-up forms, and laboratory reports) will be maintained at the study site by the site PI. These records will be reviewed during the scheduled monitoring visits by the PI to ensure compliance. The site PI will be responsible for the completeness and accuracy of the study materials and the nodal site PIs will check these during their visits to the study sites. Any observations/corrective measures required will be duly communicated to the site PIs for compliance. These records will be sent to the PI at CCRH headquarters after the completion of the study or as per the requirement of the study. The data of the laboratory parameters that indicate the safety aspects of the drug will be evaluated at the end of each of the three schedules. They will be presented/submitted to the DSMB for their recommendations. The data will be analyzed after completion of the study.

### Statistical Analysis

Data analysis will be performed following the intention-to-treat/per-protocol method. The safety and immunological parameters will be analyzed at each of the seven time points and comparisons will be made between the subjects and groups. Additional analyses (eg, subgroup analyses according to age, sex, and perceived risk categories) will also be performed based on the evolving trends from the analysis. A trial statistician will be engaged for the statistical analysis of the clinical trial.

### Ethics Considerations

#### Research Ethics Approval

The Scientific Advisory Board and the Central Ethics Committee of the CCRH have approved the study protocol and the amendments (6-2/2020-2L/CCRH/Tech./27thEC/4656; August 16, 2022). The study will be conducted in accordance with the Declaration of Helsinki and the standards of Good Clinical Practice in India. CCRH will provide insurance cover in the trial for the said study duration, according to the terms finalized under clinical trial cover with the identified insurance firm.

#### Protocol Amendments

The investigator has to meet the study requirements as specified in the protocol. Protocol amendments are possible only in exceptional cases (eg, where the health or well-being of the participant is affected) and only after approval by the Ethics Committee. Every amendment must be justified in writing and signed by all those concerned. Every amendment will be notified to the CTRI. Circulating protocols will be numbered and their distribution list will be maintained. The PI will update all circulating protocols by adding the amendment.

#### Informed Consent

Individuals above the age of 18 years will be considered eligible for consent. The site PI will obtain voluntary consent from the participants before the screening test after explaining the study. The participants will be provided with the Participant Information Sheet describing the study details and will voluntarily sign the consent form if they agree to take the intervention. The site PI must also countersign the consent form at each study site.

#### Confidentiality

The investigator will inform the participants that all trial results recorded will be treated in strict confidence. During documentation and analysis, the participants will only be identified by their subject code and unique identifier number. In contrast, the name of the participant and any personal data of the participants will be stored as per data protection regulations.

#### Dissemination Policy

The investigators will communicate trial results to participants, health care professionals, the public, and other relevant groups (via publication, reporting in results databases, or other data-sharing arrangements) after the completion of the study. However, no information based on unjustified claims or the findings of interim analysis would be communicated in any form.

#### Auditing and Inspecting

The PI will permit study-related monitoring, audits, and inspections by the DSMB constituted for the study, Ethical Committee, or government regulatory bodies and make available all study-related documents (eg, source documents, regulatory documents, data collection instruments, study data). The PI will also ensure the capability to inspect applicable study-related facilities (eg, pharmacy, diagnostic laboratory).

## Results

The CCRH, Ministry of Ayush, Government of India, funded the project in September 2022. Further, collaborations with the Department of Homoeopathy, Government of Kerala, were initiated, and the study was rolled out on January 25, 2023. As of April 3, 2023, the enrollment has been completed and the immunological assays will be conducted at DBT-THSTI, Faridabad, India.

## Discussion

The COVID-19 pandemic has become widespread worldwide and severely impacted the human population. Vaccination efforts have increased exponentially in the past 2 years to protect the vulnerable population, mainly through emergency clearances. According to the vaccine study literature, adverse effects have always been part of mass vaccination strategies across the globe. According to the WHO, in the case of side effects of inactivated virus–based vaccines, the most common local and systemic adverse reactions are injection site reactions, fatigue, fever, headache, and allergic dermatitis, which are self-limiting [34,35]. With the emerging evidence of breakthrough infections and reduced protection against the Delta variant of SARS-CoV-2, an effective preventive therapeutic strategy still needs to be identified.

During the COVID-19 pandemic, the homeopathic medicine AA30CH was widely distributed to the 10.6 million Indian population as a prophylactic. The data obtained from 580,000 individuals were analyzed to conclude that preventive consumption significantly reduced the risk of contracting COVID-19 in high-risk groups [26]. A prospective parallel cluster cohort study conducted by Nayak et al [28] reported the protective effect of AA30CH against laboratory-confirmed COVID-19 cases of 74.40%. Interestingly, this study also reported AA30CH treatment as an adjunct to standard treatment in COVID-19 patients [28]; however, the current protocol is the first investigation exploring the immunological efficacy and safety of AA30CH in an RCT. Different aspects in clinical trials with homeopathic drugs were previously explored, where the prominence of placebo-controlled RCTs in homeopathy was critically reviewed [1]. The medications used in those RCTs of homeopathy were mostly prepared as per the guidelines laid out in pharmacopoeia of the respective countries; however, several recommendations in the design and conduct of trials on homeopathic medicinal products were recently discussed [2]. In a previously published RCT in homeopathy conducted in France, the intervention was prepared according to the French National Pharmacopoeia standards, where the placebo was identical pillules containing only unmedicated pharmaceutical-grade ethanol [3]. The preparation for both the intervention and placebo in this study will be similar to that adopted in previous studies; however, we will adhere to the standards mentioned in the Homoeopathic Pharmacopoeia of India.

Several previous studies evaluated the immunological responses to COVID-19 infection and the efficacy of vaccine candidates [36,37]. The strategies toward these immunologic responses to develop candidate vaccines were vastly explained [38,39]. Immunological considerations to develop vaccine candidates were reported [40]; however, no such system was established for any alternative system of medicines, especially for homeopathy. This study intends to explicitly reveal the protective effect of AA30CH against COVID-19 by investigating the possible immune mechanisms that might play a role in its prophylactic action. The current protocol represents the first such evaluation, including immunological phenotyping; determining the antigen density; and identifying the variation in immunological markers and lymphocyte subsets CD3, CD4, CD8, CD24, CD27, CD-38, CD4 IFN-γ, CD4 CD17, CD4 CD25 (activated T lymphocytes), T cells, B cells, DCs (mature and immature), and NK cells.

The current protocol also investigates the expression analysis of 84 vital genes of interest related to the cellular pathways associated with innate and adaptive immune responses. This will be studied in detail using a well-established RT-PCR array profiler previously used for several other viruses [41-45]. The array selected for this study includes genes related to the IL-1R and TLR signaling pathways and genes involved in the sensing of pathogens. Genes related to the host defense response are also represented on this array, including the acute-phase response, complement activation, inflammatory responses, and antiviral humoral response. NK cells are reported to be vital to lyse virus-infected cells, thereby controlling the viral infection and cellular damage [38]. This protocol also aims to investigate the expression profiles of vital genes involved in the human NK cell responses, which may further reveal the possible effect of AA30CH on viral sensing and neutralization.

As mainly overseeing the primary objective of immunological efficacy and safety of AA30CH, this study also aims to assess the safety of AA30CH in terms of the results of LFTs, RFTs, CBCs, and peripheral smears between the intervention and placebo groups. Previously, the acute and subacute toxicity of AA30CH were investigated in an animal model and no adverse events or organ-related toxic effects were reported [46]. This study would also evaluate these findings in an RCT, which may support establishing the safety profile of AA30CH that could further open new vistas of research for an effective prophylactic therapy against SARS-CoV2 infection.

The timely, detailed investigation of the immunopharmacology of drug candidates is challenging [47,48]. In searching for therapeutic strategies against the COVID-19 pandemic, these essential investigations may be limited to a preliminary analysis or primary data obtained due to the pressing priority. Based on the objectives, our study is limited to the immunological responses of AA30CH against COVID-19 in healthy individuals; however, when AA30CH is mass distributed in an ongoing COVID-19 transmissible zone, a detailed investigation on the prevalence of COVID-19 incidence may provide more insights into its prophylactic efficacy.

In conclusion, the current RCT primarily emphasizes the immunological efficacy of AA30CH as a COVID-19 prophylaxis. Secondarily, the hematologic, hepatic, and renal toxicity evaluation of AA30CH would provide a comprehensive understanding of its use in healthy individuals; however, further directions on these parameters in comorbid conditions still need investigation.

## Appendix

Multimedia Appendix 1 Reverse transcription-polymerase chain reaction array gene list.

Multimedia Appendix 2 Peer-review reports from Central Council for Research in Homeopathy.

Multimedia Appendix 3 Peer-review reports from Central Council for Research in Homeopathy.

## Abbreviations

AA30CH: Arsenicum album 30CH
CBC: complete blood count
CCRH: Central Council for Research in Homoeopathy
CRF: case record form
CTRI: Clinical Trials Registry-India
DBT-THSTI: Department of Biotechnology, Translational Health Science and Technology Institute
DC: dendritic cell
DSMB: Data and Safety Monitoring Board
IFN: interferon
IL-1R: interleukin 1 receptor
LFT: liver function test
NCDC: National Centre for Disease Control
NHRIMH: National Homoeopathy Research Institute in Mental Health
NK: natural killer
PBMC: peripheral blood mononuclear cell
PI: principal investigator
RCT: randomized controlled trial
RFT: renal function test
RT-PCR: reverse transcription-polymerase chain reaction
SAE: severe adverse event
SPIRIT: Standard Protocol Items: Recommendations for Interventional Trials
TLR: Toll-like receptor
TPR: test positivity rate
WHO: World Health Organization

## Data Availability Statement

No data are associated with the protocol. The details of the protocol can also be accessed from the Clinical Trials Registry-India.
