# Supplementary material for: Immunological Responses of Arsenicum album 30CH to Combat COVID-19: Protocol for a Double-Blind, Randomized, Placebo-Controlled Clinical Trial in the Pathanamthitta District of Kerala
Source: JMIR Res Protoc. 2023 Oct 16;12:e48479. doi: 10.2196/48479 (PMC10616730; doi:10.2196/48479)
Supplement: Multimedia Appendix 1 [file resprot_v12i1e48479_app1.pdf]

Supplementary Table. 1. PCR Array gene list

Position	UniGene	GenBank	Symbol	Description
A01	Hs.507080	NM_001639	APCS	Amyloid P component, serum
A02	Hs.529053	NM_000064	C3	Complement component 3
A03	Hs.2490	NM_033292	CASP1	Caspase 1, apoptosis-related cysteinepeptidase (interleukin 1, beta, convertase)
A04	Hs.303649	NM_002982	CCL2	Chemokine (C-C motif) ligand 2
A05	Hs.514821	NM_002985	CCL5	Chemokine (C-C motif) ligand 5
A06	Hs.184926	NM_005508	CCR4	Chemokine (C-C motif) receptor 4
A07	Hs.450802	NM_000579	CCR5	Chemokine (C-C motif) receptor 5
A08	Hs.46468	NM_004367	CCR6	Chemokine (C-C motif) receptor 6
A09	Hs.113222	NM_005201	CCR8	Chemokine (C-C motif) receptor 8
A10	Hs.163867	NM_000591	CD14	CD14 molecule
A11	Hs.631659	NM_000616	CD4	CD4 molecule
A12	Hs.472860	NM_001250	CD40	CD40 molecule, TNF receptor superfamily member 5
B01	Hs.592244	NM_000074	CD40LG	CD40 ligand
B02	Hs.838	NM_005191	CD80	CD80 molecule
B03	Hs.171182	NM_006889	CD86	CD86 molecule
B04	Hs.85258	NM_001768	CD8A	CD8a molecule
B05	Hs.709456	NM_000567	CRP	C-reactive protein, pentraxin-related
B06	Hs.1349	NM_000758	CSF2	Colony stimulating factor 2 (granulocyte-macrophage)
B07	Hs.632586	NM_001565	CXCL10	Chemokine (C-X-C motif) ligand 10
B08	Hs.198252	NM_001504	CXCR3	Chemokine (C-X-C motif) receptor 3
B09	Hs.190622	NM_014314	DDX58	DEAD (Asp-Glu-Ala-Asp) box polypeptide 58
B10	Hs.2007	NM_000639	FASLG	Fas ligand (TNF superfamily, member 6)
B11	Hs.247700	NM_014009	FOXP3	Forkhead box P3
B12	Hs.524134	NM_002051	GATA3	GATA binding protein 3
C01	Hs.181244	NM_002116	HLA-A	Major histocompatibility complex, class I, A
C02	Hs.650174	NM_005516	HLA-E	Major histocompatibility complex, class I, E
C03	Hs.643447	NM_000201	ICAM1	Intercellular adhesion molecule 1
C04	Hs.37026	NM_024013	IFNA1	Interferon, alpha 1
C05	Hs.529400	NM_000629	IFNAR1	Interferon (alpha, beta and omega) receptor 1
C06	Hs.93177	NM_002176	IFNB1	Interferon, beta 1, fibroblast
C07	Hs.856	NM_000619	IFNG	Interferon, gamma
C08	Hs.520414	NM_000416	IFNGR1	Interferon gamma receptor 1

C09	Hs.193717	NM_000572	IL10	Interleukin 10
C10	Hs.845	NM_002188	IL13	Interleukin 13
C11	Hs.41724	NM_002190	IL17A	Interleukin 17A
C12	Hs.83077	NM_001562	IL18	Interleukin 18 (interferon-gamma-inducing factor)
D01	Hs.1722	NM_000575	IL1A	Interleukin 1, alpha
D02	Hs.126256	NM_000576	IL1B	Interleukin 1, beta
D03	Hs.701982	NM_000877	IL1R1	Interleukin 1 receptor, type I
D04	Hs.89679	NM_000586	IL2	Interleukin 2
D05	Hs.98309	NM_016584	IL23A	Interleukin 23, alpha subunit p19
D06	Hs.73917	NM_000589	IL4	Interleukin 4
D07	Hs.2247	NM_000879	IL5	Interleukin 5 (colony-stimulating factor, eosinophil)
D08	Hs.654458	NM_000600	IL6	Interleukin 6 (interferon, beta 2)
D09	Hs.624	NM_000584	IL8	Interleukin 8
D10	Hs.522819	NM_001569	IRAK1	Interleukin-1 receptor-associated kinase 1
D11	Hs.75254	NM_001571	IRF3	Interferon regulatory factor 3
D12	Hs.166120	NM_001572	IRF7	Interferon regulatory factor 7
E01	Hs.172631	NM_000632	ITGAM	Integrin, alpha M (complement component 3 receptor 3 subunit)
E02	Hs.656213	NM_004972	JAK2	Janus kinase 2
E03	Hs.660766	NM_015364	LY96	Lymphocyte antigen 96
E04	Hs.524579	NM_000239	LYZ	Lysozyme
E05	Hs.431850	NM_002745	MAPK1	Mitogen-activated protein kinase 1
E06	Hs.138211	NM_002750	MAPK8	Mitogen-activated protein kinase 8
E07	Hs.499674	NM_000242	MBL2	Mannose-binding lectin (protein C) 2, soluble
E08	Hs.458272	NM_000250	MPO	Myeloperoxidase
E09	Hs.517307	NM_002462	MX1	Myxovirus (influenza virus) resistance 1, interferon-inducible protein p78 (mouse)
E10	Hs.82116	NM_002468	MYD88	Myeloid differentiation primary response gene (88)
E11	Hs.654408	NM_003998	NFKB1	Nuclear factor of kappa light polypeptide gene enhancer in B-cells 1
E12	Hs.81328	NM_020529	NFKBIA	Nuclear factor of kappa light polypeptide gene enhancer in B-cells inhibitor, alpha
F01	Hs.159483	NM_183395	NLRP3	NLR family, pyrin domain containing 3
F02	Hs.405153	NM_006092	NOD1	Nucleotide-binding oligomerization domain containing 1
F03	Hs.592072	NM_022162	NOD2	Nucleotide-binding oligomerization domain containing 2
F04	Hs.73958	NM_000448	RAG1	Recombination activating gene 1
F05	Hs.256022	NM_005060	RORC	RAR-related orphan receptor C

F06	Hs.591607	NM_000578	SLC11A1	Solute carrier family 11 (proton-coupled divalent metal ion transporters), member 1
F07	Hs.642990	NM_007315	STAT1	Signal transducer and activator of transcription 1, 91kDa
F08	Hs.463059	NM_003150	STAT3	Signal transducer and activator of transcription 3 (acute-phase response factor)
F09	Hs.80642	NM_003151	STAT4	Signal transducer and activator of transcription 4
F10	Hs.524518	NM_003153	STAT6	Signal transducer and activator of transcription 6, interleukin-4 induced
F11	Hs.272409	NM_013351	TBX21	T-box 21
F12	Hs.29344	NM_182919	TICAM1	Toll-like receptor adaptor molecule 1
G01	Hs.654532	NM_003263	TLR1	Toll-like receptor 1
G02	Hs.519033	NM_003264	TLR2	Toll-like receptor 2
G03	Hs.657724	NM_003265	TLR3	Toll-like receptor 3
G04	Hs.174312	NM_138554	TLR4	Toll-like receptor 4
G05	Hs.604542	NM_003268	TLR5	Toll-like receptor 5
G06	Hs.662185	NM_006068	TLR6	Toll-like receptor 6
G07	Hs.659215	NM_016562	TLR7	Toll-like receptor 7
G08	Hs.660543	NM_138636	TLR8	Toll-like receptor 8
G09	Hs.87968	NM_017442	TLR9	Toll-like receptor 9
G10	Hs.241570	NM_000594	TNF	Tumor necrosis factor
G11	Hs.591983	NM_004620	TRAF6	TNF receptor-associated factor 6
G12	Hs.75516	NM_003331	TYK2	Tyrosine kinase 2
H01	Hs.520640	NM_001101	ACTB	Actin, beta
H02	Hs.534255	NM_004048	B2M	Beta-2-microglobulin
H03	Hs.592355	NM_002046	GAPDH	Glyceraldehyde-3-phosphate dehydrogenase
H04	Hs.412707	NM_000194	HPRT1	Hypoxanthine phosphoribosyltransferase 1
H05	Hs.546285	NM_001002	RPLP0	Ribosomal protein, large, P0
H06	N/A	SA_00105	HGDC	Human Genomic DNA Contamination
H07	N/A	SA_00104	RTC	Reverse Transcription Control
H08	N/A	SA_00104	RTC	Reverse Transcription Control
H09	N/A	SA_00104	RTC	Reverse Transcription Control
H10	N/A	SA_00103	PPC	Positive PCR Control
H11	N/A	SA_00103	PPC	Positive PCR Control
H12	N/A	SA_00103	PPC	Positive PCR Control
