# Supplementary material for: Immunological Responses of Arsenicum album 30CH to Combat COVID-19: Protocol for a Double-Blind, Randomized, Placebo-Controlled Clinical Trial in the Pathanamthitta District of Kerala
Source: JMIR Res Protoc. 2023 Oct 16;12:e48479. doi: 10.2196/48479 (PMC10616730; doi:10.2196/48479)
Supplement: Multimedia Appendix 2 [file resprot_v12i1e48479_app2.pdf]

www.ccrhindia.nic.in ccrhindia@gmail.com Tel: 011-28524415, 1162, 5523, 5749 Fax-011-28521060

केंद्रीय होम्योपैथी अनुसन्धान परिषद्

(स्वायत्त्य निकाय आयुष मंत्रालय, भारत सरकार)

CENTRAL COUNCIL FOR RESEARCH IN HOMOEOPATHY

जवाहर लाल नेहरु भारतीय चिकित्सा एवं होम्योपैथी अनुसन्धान भवन

Jawahar Lal Nehru Bhartiya Chikitsa Avum Homoeopathy Anusandhan Bhawan

61-65, Institutional Area, Opp. D-Block, Janakpuri, New Delhi – 110058

G20 भारत 2023 INDIA

वसुधैव कुटुम्बकम्

ONE EARTH · ONE FAMILY · ONE FUTURE

F.No. 1-37/2021-22/CCRH/CR/Tech/AA_30 Immunology/Pf-3 505 Date: 12/05/23

To
The Editor
JMIR Research Protocols

Sub: Peer review comments for the grant-funded study protocol -reg.

Sir/Madam,

The following experts have peer-reviewed the grant-funded (sanction order no. 620/2022-23) protocol of the study titled *'Immunological responses of Arsenicum album 30C to combat COVID-19: A double-blind, randomized, placebo-controlled clinical trial in the Pathanamthitta district of Kerala'* and shared their comments in e-mail:

1. **Prof. (Dr.) Kanjaksha Ghosh**, MD (Med), DNB (Haem), MAMS (Path), MRCPI (Ireland), MRCP (UK), MRCPath (Lond: Haem), FRCP (Glasgow), FRCPath (Lond), FAMS, FACP (USA), FICP, FISHTM, FNASc, Former Director NIIH (National Institute of Immunohaematology, Maharashtra, India)
   E-mail ID: kanjakshaghosh@hotmail.com
2. **Dr. Bhaskar Saha**, Ph.D., Scientist 'G', National Centre for Cell Science, Department of Biotechnology (DBT-NCCS), Government of India
   E-mail ID: bhaskar211964@yahoo.com
3. **Dr. Atul Juneja**, Ph.D. Statistics, Former Scientist-F, National Institute of Medical Statistics, Indian Council of Medical Research, Department of Health Research, Ministry of Health and Family Welfare, Government of India
   E-mail ID: atul_juneja@hotmail.com

The original peer-review comments are also attached for your reference so that the paper may be made eligible for a 50% APF waiver.

Thanks & Regards

12.05.2023

Dr. Subhash Kaushik,
Director General, CCRH

Immunology of Arsenic album 30C RCT : Review of protocol - reg.

Dr.Suhana Azis <sui.azis@gmail.com> Thu, Nov 18, 2021 at 9:49 PM
To: Kanjaksha Ghosh <kanjakshaghosh@hotmail.com>
Cc: Anil Kumar Khurana <anilnaman@rediffmail.com>, Praveen Oberai <oberai.praveen@gmail.com>

Respected Sir,

Thank you very much for reviewing our protocol. As per our telephonic conversation held on 11th November 2021, we have incorporated your valuable comments in the revised version of the project protocol (*Protocol version_2.0_18_11_2021)*. The revised protocol with point-by-point responses to the queries raised is provided below.

Q1: In RCT, the parameters like T-cell, B cell count do not give any functional measure of immune activity by AA30. The change in count may even be attributed to the physiological re-distribution of T-cell, B-cell, and the results of the study may not be appreciated. Hence suggested adding parameters like NK cell count which relates to the functional activity of the immune system and are virus-specific.

A1: We have included NK cells counts in the protocol. In addition, the PCR array experiments in the protocol will be used to characterize 84 genes involved in the innate and adaptive immune responses. Some of the genes in this list provided in the protocol are previously reported to contribute to the SARS-CoV-2 infection. Moreover, in case of any specific requirement, gene lists will be customized specific, and to be incorporated in the revised protocol. To characterize NK cells as suggested, gene expression profiling of the human natural killer cell responses will be also in consideration, as mentioned previously (Campbell et. al 2015).

Q2: Regarding the two treatment arms to be kept; Vaccinated and non-vaccinated.
A2: We have included the two arms as suggested in the revised protocol. Both vaccinated and unvaccinated subjects will be given Ars Alb 30C or placebo. Using a subgroup analysis, data will be compared and represented in a pie chart explaining the immune responses as vaccinated v/s unvaccinated.

Q3: Regarding in vitro analysis

- Mentioned about what if co-cultures of Monocyte/lymphocytes are added?
- Including live virus stocks instead of pseudovirus in the cultures.

A3: The enclosed protocol is aimed only for conducting the RCT and funding is requested for the same only. The in vitro studies are currently ongoing at THSTI, Faridabad , Under DBT(under an MoU with the Department of Homoeopathy, Kerala State). The PI of this proposed project (RCT) is one of the Co-PIs of the in vitro studies and your valuable suggestion was incorporated in the revised version of in vitro protocol. Further to our discussions with the PI (in vitro studies), the co-culture experiments were planned to be conducted with live SARS-CoV-2 under BSL-3 conditions with the most potent dilution of Arsenic Album that will be identified with the pseudovirus infection studies. Currently, we proceed with the amendments in the MoU and are further in the process of budgeting for additional experiments. The

experiments will be conducted at THSTI (coordinated by State Homoeopathy Institute for Interdisciplinary Research and Training - SHIIRT, Government of Kerala).

Q4: Suggestion to send the protocol to Dr. Bhaskar Saha, NCCS for more comments.
A4: The protocol was already sent to Dr. Bhaskar Saha and he has recommended the protocol for funding, which was already communicated with DG-CCRH.

Request you to kindly review the attached revised protocol (*Protocol version_2.0_18_11_2021)* and provide your further opinion on the same.

Thanks & Regards,
Dr. Suhana P . Azis, MD
Research Officer (Homoeopathy),
Central Council for Research in Homoeopathy(CCRH)
Ministry of AYUSH,
Government of India
http://ccrhindia.nic.in/

HOMOEOPATHIC CLINICAL CASE REPOSITORY
HCCR

https://hccr.ccrhindia.in/en/

Protocol version_2.0_18_11_2021 (1).pdf
1038K

Gmail Dr Suhana Azis <sui.azis@gmail.com>

Immunology of Arsenic album 30C RCT : Review of protocol - reg.

Kanjaksha Ghosh <kanjakshaghosh@hotmail.com> Sat, Nov 20, 2021 at 7:36 PM
To: "Dr.Suhana Azis" <sui.azis@gmail.com>

Dr Suhana,
I have gone through the protocol it is quite comprehensive. However your PCR array has to be transcriptomic ie based on mRNA . The mRNA from the cells needs to be converted into cDNA and quantitated for specific genes. This should be mentioned. You should preserve an aliquot of cDNA so obtained for future studies.
You have not mentioned any marker on Dendritic cells kindly include that. There are mature an immature dendritic cells both needs to be quantitated.
CD4 cells should also be tested for Cd4 interferon gamma and CD4 CD17 as well asCD4 CD25 as activated T lymphocytes.
In case no changes in cell number is found at least measure antigen Density on flow cytometry. this can be easily done in the sample without any extra expenditure.
I have told you try to include some vaccinated people and give them Ars alb.. Following that measure COVID19 antibody response between two groups. In fact anti covid 19 antibody should be measured in all this will ive an indication of not only strength of the antibody on medication but also will tell us how many ptients got subclinical infection in both groups.
One of the problem with the proposal is that it is not detailed enough on its techniques.
If possible phagocytic activity should also be measured with ars album therapy.
I need not see the project again. Include the relevant changes that is possible and submit.

Prof. (Dr) Kanjaksha Ghosh
MD (Med), DNB (Haem), MAMS (Path), MRCPI (Ireland),
MRCP (UK), MRCPath (Lond:Haem), FRCP (Glasgow),
FRCPath (Lond), FAMS, FACP (USA), FICP, FISHTM, FNASc

Former Director NIIH (National Institute of Immunohaematology)
13th floor, New Multi-storeyed Building,
KEM Hospital Campus, Parel,
Mumbai, Maharashtra - 400012

From: Dr.Suhana Azis <sui.azis@gmail.com>
Sent: Thursday, November 18, 2021 9:49 PM
To: Kanjaksha Ghosh <kanjakshaghosh@hotmail.com>
Cc: Anil Kumar Khurana <anilnaman@rediffmail.com>; Praveen Oberai

Gmail

Dr Suhana Azis <sui.azis@gmail.com>

Immunology of Arsenic album 30C RCT : Review of protocol - reg.

Dr.Suhana Azis <sui.azis@gmail.com> Sat, Nov 20, 2021 at 9:20 PM
To: Kanjaksha Ghosh <kanjakshaghosh@hotmail.com>
Cc: Anil Kumar Khurana <anilnaman@rediffmail.com>, Praveen Oberai <oberai.praveen@gmail.com>

Respected Sir,

Thankyou for your detailed response. These will help us in improving the protocol to a great extent. Will do the needful and make our submission for funding.

[Quoted text hidden]

--

[Quoted text hidden]

Gmail

Dr Suhana Azis <sui.azis@gmail.com>

Review of research protocol titled 'Immunological responses of Arsenicum album 30C to overcome COVID 19: A double-blind randomized, placebo-controlled clinical trial in the Pathanamthitta district of Kerala' on an urgent basis – reg.

Dr.Suhana Azis <sui.azis@gmail.com> Fri, Nov 5, 2021 at 4:34 PM
To: bhaskar211964@yahoo.com, sahab@nccs.res.in
Cc: Anil Kumar Khurana <anilnaman@rediffmail.com>, Praveen Oberai <oberai.praveen@gmail.com>
Bcc: Sreekanth Gopinathan Pillai <sreekanthsreebhavan@gmail.com>

Sir,

As per the advisory brought out by the Ministry of Ayush on recommendations of the Scientific Advisory Board (SAB) of CCRH, the homeopathic medicine *Arsenicum album 30C* has been distributed as a prophylactic medicine to COVID-19, to over one crore Indian population. Till date , CCRH has conducted population based clinical trials and field studies to understand the protective effect of *Arsenicum album 30C* but the immunological aspects after intake of the medicine have not been studied yet. The research protocol mentioned in the subject above has been proposed to explore the possible immunological responses of *Arsenicum album 30C* to COVID 19 in a double-blind randomized controlled clinical trial design, to build scientific evidence and establish the prophylactic role of the medicine.

As per directions of competent authority , you are kindly requested to urgently review the enclosed protocol and give your expert comments within **three days** in lieu of the looming threat of a third wave of the pandemic and to be further placed in scientific committees for needful approvals.

Kindly acknowledge receipt of this email.

Enclosed: 1. Letter CCRH
2. RCT protocol for review as pdf file. Kindly add comments in the same.
3. Supplementary invitro protocol for reference

--

Thanks & Regards,
Dr. Suhana P . Azis, MD
Research Officer (Homoeopathy),
Central Council for Research in Homoeopathy(CCRH)
Ministry of AYUSH,
Government of India
http://ccrhindia.nic.in/

HOMOEOPATHIC CLINICAL CASE REPOSITORY
HCCR

https://hccr.ccrhindia.in/en/

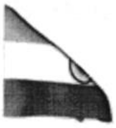

---

3 attachments

Letter_Dr_Saha_05_11_21.pdf
471K

AA_30_RCT_Review_05_11_21.pdf
1608K

Final_invitro_Thsti.pdf
2708K

Gmail

Dr Suhana Azis <sui.azis@gmail.com>

Review of research protocol titled 'Immunological responses of Arsenicum album 30C to overcome COVID 19: A double-blind randomized, placebo-controlled clinical trial in the Pathanamthitta district of Kerala' on an urgent basis – reg.

bhaskar saha <bhaskar211964@yahoo.com> Tue, Nov 9, 2021 at 4:36 PM
To: sahab@nccs.res.in, "Dr.Suhana Azis" <sui.azis@gmail.com>
Cc: Anil Kumar Khurana <anilnaman@rediffmail.com>, Praveen Oberai <oberai.praveen@gmail.com>

Dear Dr Azis,
The proposal is detailed. The rationale, study objectives, experimental approach, sample groups and sample analysis are sciemtifically described. So, I am inclined to recommend this proposal for funding.
Thank you. With best regards,
Bhaskar Saha

[Quoted text hidden]

Gmail **Dr Suhana Azis <sui.azis@gmail.com>**

Statistical Discussion on research proposal on 'Immunology of Ars alb 30C' - reg.

Atul Juneja <atul_juneja@hotmail.com> Thu, Dec 2, 2021 at 5:03 PM
To: "Dr.Suhana Azis" <sui.azis@gmail.com>, "shajikumar2016@gmail.com" <shajikumar2016@gmail.com>

As discussed, I am enclosing the paragraph on issue of sample size, for your consideration. You may change accooring to your suited terminology the medical contents
Atul Juneja

The study is aimed at evaluating immunogenic responces of Arsenic Album. The study is based on earlier encouraging leads provided through the community studies on role of Arsenic Al. in prevention of Covid -19. There are number of parameters which are in consideration for evaluation of immunogenic response. As regards computation of sample size , based on the literature and experts 'opinion it is proposed to consider somelimited immunogenic parameters which are more relevant. Since in view of very limited availability of literature it would be quite challenging to hypothesizethe assumption. Based on the preliminary study carried out in Kerala, the experts were of the opinion that CD4 and CD 8 count would be considered for estimating the sample size. Although the study had a small sample size the study did provide leads for the large studies. It was observed most of the subjects showed an elevation in CD4 and CD 8 count with intervention of interest. Based on these results, It was felt that an increase of 20% in CD4 count and an increase of 15% in CD8 count could be considered as the favorable outcome of interest. Based on the conservative approach it was decided that it could be reasonable to assume that the 50% of the population would express an increase in CD4 and CD 8 to the desired levels mentioned above. Whereas in the control group where there was no active drug given, there could be a natural increase in above mentioned immunogenic parameters in 30 percent of the population.

Assuming type 1 error of 5 percent and power of 80 percent and above-mentioned proportions it was estimated that there would be 93 cases which would be needed in each case and control group. This could be escalated based on attrition due to loss in follow-up. If this loss to follow is considered at 20% then a total of 112 cases per arm would need to be recruited for the study.

From: Dr.Suhana Azis <sui.azis@gmail.com>
Sent: Friday, November 26, 2021 7:04 PM
To: atul_juneja@hotmail.com <atul_juneja@hotmail.com>

Cc: Sreekanth Gopinathan Pillai <sreekanthsreebhavan@gmail.com>; shaji kumar <shajikumar2016@gmail.com>; Dr. Oriparambil Sivaraman Nirmal Ghosh <nirmalgvr@protonmail.ch>; Suhana Azis <sui.azis@gmail.com>
Subject: Statistical Discussion on research proposal on 'Immunology of Ars alb 30C' - reg.

[Quoted text hidden]
