# Supplementary material for: Immunological Responses of Arsenicum album 30CH to Combat COVID-19: Protocol for a Double-Blind, Randomized, Placebo-Controlled Clinical Trial in the Pathanamthitta District of Kerala
Source: JMIR Res Protoc. 2023 Oct 16;12:e48479. doi: 10.2196/48479 (PMC10616730; doi:10.2196/48479)
Supplement: Multimedia Appendix 3 [file resprot_v12i1e48479_app3.pdf]

www.ccrhindia.nic.in
Fax: 011-28521060

Tel: 011-28524415,1162,5523,5749
EPABX Board: 011-28525831,852,862

केंद्रीय होम्योपैथिक अनुसन्धान परिषद्
(स्वायत्त निकाय आयुष मंत्रालय, भारत सरकार)
CENTRAL COUNCIL FOR RESEARCH IN HOMOEOPATHY
(An Autonomous Body of Ministry of AYUSH, Govt. of India)
जवाहर लाल नेहरु भारतीय चिकित्सा एवं होमियोपैथी अनुसन्धान भवन
Jawahar Lal Nehru Bhartiya Chikitsa Avum Homoeopathy Anusandhan Bhawan
61-65 संस्थागत क्षेत्र, डी-ब्लॉक के सामने, जनकपुरी, नई दिल्ली - 110058
61-65, Institutional Area, Opp. D-Block, Janakpuri, New Delhi – 110058

150 YEARS OF CELEBRATING THE MAHATMA

F. No.1-37/2021-22/CCRH/CR/Tech/AA30-Immunology 7525 Dated: 05/11/2021

To

Prof. (Dr) Kanjaksha Ghosh,
Former Director, National Institute of Immunohematology (NIIH),
13th Floor, New multi storeyed building, KEM Hospital Campus, Parel,
Mumbai, Maharashtra

Sub: Review of research protocol titled 'Immunological responses of *Arsenicum album 30C* to overcome COVID 19: A double-blind randomized, placebo-controlled clinical trial in the Pathanamthitta district of Kerala' on an urgent basis – reg.

Sir,

As you are aware , based on the advisory brought out by the Ministry of Ayush on recommendations of the Scientific Advisory Board (SAB) of CCRH, the homeopathic medicine *Arsenicum album 30C* has been distributed as a prophylactic medicine to COVID-19, to over one crore Indian population. Till date , CCRH has conducted population based clinical trials and field studies to understand the protective effect of *Arsenicum album 30C* but the immunological aspects after intake of the medicine have not been studied yet. The research protocol mentioned in the subject above has been proposed to explore the possible immunological responses of *Arsenicum album 30C* to COVID 19 in a double-blind randomized controlled clinical trial design, to build scientific evidence and establish the prophylactic role of the medicine.

You are kindly requested to urgently review the enclosed protocol and give your expert comments within three days in lieu of the looming threat of a third wave of the pandemic and to be further placed in scientific committees for needful approvals.

An honorarium will be paid as per rules and approval of the competent authority.

Thanking you,

Yours faithfully,

Poberai

Dr. Praveen Oberai
Deputy Director General (I/c)

www.ccrhindia.nic.in
Fax: 011-28521060

Tel: 011-28524415,1162,5523,5749
EPABX Board: 011-28525831,852,862

केंद्रीय होम्योपैथिक अनुसन्धान परिषद्
(स्वायत्त निकाय आयुष मंत्रालय, भारत सरकार)
CENTRAL COUNCIL FOR RESEARCH IN HOMOEOPATHY
(An Autonomous Body of Ministry of AYUSH, Govt. of India)
जवाहर लाल नेहरु भारतीय चिकित्सा एवं होमियोपैथी अनुसन्धान भवन
Jawahar Lal Nehru Bhartiya Chikitsa Avum Homoeopathy Anusandhan Bhawan
61-65 संस्थागत क्षेत्र, डी-ब्लॉक के सामने, जनकपुरी, नई दिल्ली - 110058
61-65, Institutional Area, Opp. D-Block, Janakpuri, New Delhi – 110058

150 YEARS OF CELEBRATING THE MAHATMA

F. No.1-37/2021-22/CCRH/CR/Tech/AA30-Immunology 7524 Dated: 05/11/2021

To

Dr. Bhaskar Saha, Scientist E
National Centre for Cell Science
NCCS Complex, Pune University Campus, Ganeshkhind
Pune - 411 007, Maharashtra

Sub: Review of research protocol titled 'Immunological responses of *Arsenicum album 30C* to overcome COVID 19: A double-blind randomized, placebo-controlled clinical trial in the Pathanamthitta district of Kerala' on an urgent basis – reg.

Sir,

As per the advisory brought out by the Ministry of Ayush on recommendations of the Scientific Advisory Board (SAB) of CCRH, the homeopathic medicine *Arsenicum album 30C* has been distributed as a prophylactic medicine to COVID-19, to over one crore Indian population. Till date , CCRH has conducted population based clinical trials and field studies to understand the protective effect of *Arsenicum album 30C* but the immunological aspects after intake of the medicine have not been studied yet. The research protocol mentioned in the subject above has been proposed to explore the possible immunological responses of *Arsenicum album 30C* to COVID 19 in a double-blind randomized controlled clinical trial design, to build scientific evidence and establish the prophylactic role of the medicine.

You are kindly requested to urgently review the enclosed protocol and give your expert comments within three days in lieu of the looming threat of a third wave of the pandemic and to be further placed in scientific committees for needful approvals.

An honorarium will be paid as per rules and approval of the competent authority.

Thanking you,

Yours faithfully,

Dr. Praveen Oberai
Deputy Director General (I/c)

From pg. 10

Sub: Review comments received from domain experts and incorporated in Immunology protocol – reg.

As directed in pre pg 9/N, the research protocol on Immunology of Ars alb 30C was reviewed by Dr. Bhaskar Saha, Virologist, NCCS, Pune & comments received on 9.11.2021 (Flag A) and Dr. Kanjaksha Ghosh, Immunologist whose comments received on 20.11.2021 (Flag B). The comments are compiled as below:

Sl. No	Name of the expert	Comments
1.	Dr. Bhaskar Saha	*Received on 09.11.2021*<br>• The proposal is detailed. The rationale, study objectives, experimental approach, sample groups and sample analysis are scientifically described.<br>• So, I am inclined to recommend this proposal for funding.
2.	Dr. Kanjaksha Ghosh	*Received on 20.11.2021*<br>• I have gone through the protocol it is quite comprehensive. However your PCR array has to be transcriptomic ie based on mRNA . The mRNA from the cells needs to be converted into cDNA and quantitated for specific genes. This should be mentioned. You should preserve an aliquot of cDNA so obtained for future studies.<br>• You have not mentioned any marker on Dendritic cells kindly include that. There are mature an immature dendritic cell both needs to be quantitated.<br>• CD4 cells should also be tested for Cd4 interferon gamma and CD4 CD17 as well asCD4 CD25 as activated T lymphocytes.<br>• In case no changes in cell number is found at least measure antigen Density on flow cytometry. this can be easily done in the sample without any extra expenditure.<br>• I have told you try to include some vaccinated people and give them Ars alb.. Following that, measure COVID19 antibody response between two groups. In fact anti covid 19 antibody should be measured in all this will ive an indication of not only strength of the antibody on medication but also will tell us how many patients got subclinical infection in both groups.<br>• One of the problem with the proposal is that it is not detailed enough on its techniques.<br>• If possible phagocytic activity should also be measured with ars album therapy.<br>• I need not see the project again. Include the relevant changes that is possible and submit.

Dr. Saha has recommended the protocol for funding and Dr. Ghosh has suggested minor modifications which are incorporated in the protocol. Dr. Ghosh has asked to include the relevant changes that is possible and submit further at CCRH without sending the protocol again to him. All comments by Dr. Ghosh have been incorporated as below -

- As suggested Transcriptome based PCR array is planned [i.e. RNA → cDNA → SYBR-Green Master Mix (Gene expression analysis by Real time RT-PCR) → delta^2 cT Method].
- NK cell count included .
- NK cell responses to Ars alb (gene expression analysis) included.
- Characterisation of mature and immature dendritic cells .
- Dendritic and non dendritic cell count added .
- CD4+ cells will be evaluated for CD4 IFN gamma , CD4 CD17 and CD4 CD 25 (T-lymphocyte activation).
- Antigen density will be measured by Flow cytometry .

Contd.

- COVID 19 antibody responses in *Arsenic album* will be studied on both COVID 19 vaccinated and unvaccinated subjects. Specific analysis will be conducted to characterise whether Ars alb produce any Covid 19 specific antibodies.
- Inclusion of Phagocytic activity studies will be conducted by Flow cytometry (This point is under discussion with collaborators).

For the above experiments to be included an additional budget of INR 12 lakhs have been added in the protocol. The revised *Protocol Version 2.1 dated 23.11.2021* is submitted at Flag C for guidance for further course of action.

Submitted please.

[signature] 23.11.2021
RO(SPA)

DDG(I/C)

we may place the protocol to approval of SCCR & subsequently SAB if agreed pl.

[signature] 23/11/21

DG pl.

Let the clinical prot. be submitted to SCCR committee with reduction in sample size as pilot study to reduce the financial cost of the project.

DDG on leave

RO(SPA)

[signature] 24/11

09
24/11/21 (DG)

In order to recalculate sample size and to maintain study rigour may kindly recommend a senior Statistician to see the research protocol for comments.

Submitted please.

Advice be taken from Dr Atul Juneja.

[signature] 24.11.21
RO(SPA)

DDG (I/c) (on leave) / DG pl.

[signature] 24/11

RO(SPA)

From pre pg 12

Sub: Recalculation of sample size by Dr. Atul Juneja, Scientist E ICMR - reg.

Three meetings were held on 24.11.21, 26.11.2021, 01.12.21 and 02.12.21 with Dr. Atul Juneja, Scientist E, ICMR as per directions in pre-pg 12/N to recalculate sample size of the clinical trial on 'Immunology of *Arsenic album 30C*' . In the online meeting held on 26.11.2021 (Flag A) with study team, overview of the study protocol was presented and the statistical plan was discussed in detail with Dr. Juneja. The expert was also appreciative of the study design and proposal. Based on the statistical parameters mentioned in the protocol , it was suggested that the sample size maybe recalculated based on fixing two or three parameters that would best determine the immunomodulatory effect of Arsenicum album 30C for Covid prevention.The previous pilot data had the following significant immunomodulation documented :

- The mean **CD4** count of 555.75 cells/microL in the pre-test rose to 869.68 cells/microL post test after Ars alb 30C with a mean spike of approx..**314** cells/microL post intervention.
- The mean **CD8** count of 426.56 cells/microL in the pre-test rose to 608.62 cells/microL post test after *Ars alb 30C* with a mean spike of approx..**182** cells/microL post intervention.
- The mean **CD3** count of 1016.43 cells/microL in the pre-test rose to 1535.25 cells/microL post test after *Ars alb 30C* with a mean spike of approx. **519** cells/microL post intervention.

Based on the above pilot data from the Pathanamthitta study and as per expert suggestion from Immunologist, CD4 and CD8 were the parameters selected for sample size determination. As per email **(Flag A)** communication received from Dr. Atul Juneja , the following was recommended to be added under the Statistical Plan of the study -

'A'

'The study is aimed at evaluating immunogenic responces of Arsenic Album. The study is based on earlier encouraging leads provided through the community studies on role of Arsenic Al. in prevention of Covid -19. There are number of parameters which are in consideration for evaluation of immunogenic response. As regards computation of sample size , based on the literature and experts 'opinion it is proposed to consider somelimited immunogenic parameters which are more relevant. Since in view of very limited availability of literature it would be quite challenging to hypothesise the assumption. Based on the preliminary study.carried out in Kerala, the experts were of the opinion that CD4 and CD 8 count would be considered for estimating the sample size. Although the study had a small sample size the study did provide leads for the large studies. It was observed most of the subjects showed an elevation in CD4 and CD 8 count with intervention of interest. Based on these results, It was felt that an increase of 20% in CD4 count and an increase of 15% in CD8 count could be considered as the favorable outcome of interest. Based on the conservative approach it was decided that it could be reasonable to assume that the 50% of the population would express an increase in CD4 and CD 8 to the desired levels mentioned above. Whereas in the control group where there was no active drug given, there could be a natural increase in above mentioned immunogenic parameters in 30 percent of the population.

Assuming type 1 error of 5 percent and power of 80 percent and above-mentioned proportions it was estimated that there would be 93 cases which would be needed in each case and control group. This could be escalated based on attrition due to loss in follow-up. If this loss to follow is considered at 20% then a total of 112 cases per arm would need to be recruited for the study'.

P.T.O

Sample si[illegible] 224 with 112 subject[illegible] in each a[illegible] cons[illegible] the 20% drop out[illegible] the above

'A' c/pg 14/N

S [signature] 03.12.21

we may approve the sample size of 224 cases as suggested by Dr Atul Juneja of ICMR so that the protocol can be modified accordingly

[signature] 3/12/21

[signature]

Discuss on Monday

[signature] 3/12

[signature]

SPA(S?)

[signature] 3/12/21

14 / 03/12/21 महानिदेशक

As discussed with DG Sir, the following literature were to be searched and informed to confirm the proposed sample size

a) The physiological range of CD4+ & CD48+ in Indian population.

The same has been explored and explained in pg 15/N.

Contd. to pg. 15
